# A meta-analysis of the effects of long-term oxygen therapy combined with exercise rehabilitation on exercise capacity, cardiopulmonary function, and quality of life in patients with COPD

**DOI:** 10.3389/fmed.2025.1640084

**Published:** 2025-09-22

**Authors:** Qi Muge, Suriguga, Yuqing, Aronggaowa, Taojin, Lanying Chen

**Affiliations:** ^1^Department of Geriatrics and Respiratory Medicine, Affiliated Hospital of Inner Mongolia Minzu University, Tongliao, China; ^2^School of Pharmacy, Jiangxi University of Traditional Chinese Medicine, Nanchang, Jiangxi, China; ^3^Clinical College, Inner Mongolia Minzu University, Tongliao, China; ^4^National Pharmaceutical Engineering Center for Solid Preparation of Chinese Herbal Medicine, Jiangxi University of Traditional Chinese Medicine, Nanchang, Jiangxi, China

**Keywords:** oxygen therapy, exercise rehabilitation, chronic obstructive pulmonary disease, exercise capacity, cardiorespiratory function

## Abstract

**Objective:**

To assess the effectiveness of long-term oxygen therapy (LTOT) combined with exercise rehabilitation vs. exercise rehabilitation alone in improving exercise capacity, cardiopulmonary function, and quality of life in chronic obstructive pulmonary disease (COPD) patients.

**Methods:**

A comprehensive literature search was conducted in the Chinese Biomedical Literature Database (CBM), Wanfang, China National Knowledge Infrastructure (CNKI), Cochrane Library, EMBASE, ScienceDirect, and PubMed for studies published from January 2010 to the present. Controlled clinical trials comparing oxygen therapy and/or exercise rehabilitation in COPD patients were included. Two independent reviewers extracted data and assessed risk of bias using the Cochrane Handbook (version 5.3). Meta-analysis was performed using RevMan 5.3.

**Results:**

Nine studies (*N* = 703) met inclusion criteria. Compared with the control group (CG), the combined LTOT and exercise group showed significant improvements in 6-min walk distance (6MWD), forced expiratory volume in 1 s (FEV1), and FEV1/FVC ratio (*P* < 0.05). PaO_2_ levels tended to be higher but showed substantial heterogeneity. No significant differences were observed in blood oxygen saturation, heart rate, or PaCO_2_. Quality of life significantly improved in the combined therapy group.

**Conclusion:**

LTOT combined with exercise rehabilitation is more effective than exercise alone in improving exercise capacity, pulmonary function, and quality of life in COPD patients. However, cardiac benefits remain unclear, warranting further studies.

## 1 Introduction

Chronic obstructive pulmonary disease (COPD) is a primarily characterized by symptoms such as excessive sputum production, chronic cough, and dyspnea (1). As the disease progresses, patients experience a gradual decline in physical activity tolerance and cardiopulmonary function, leading to significant challenges such as difficulty breathing during daily activities. These deteriorations can severely impact individuals' quality of life and pose life-threatening risks (2).

Long-term oxygen therapy (LTOT) is recommended for severe resting hypoxemia and improves survival; it does not routinely benefit moderate resting or isolated exertional desaturation (3). LTOT is particularly indicated for patients with severe COPD, especially those who exhibit hypoxemia even at rest (4). Specifically, according to the Global Initiative for Chronic Obstructive Lung Disease (GOLD) guidelines, LTOT is recommended for patients with resting arterial oxygen saturation (SpO_2_) ≤ 88% or partial pressure of arterial oxygen (PaO_2_) ≤ 55 mm Hg (5). Additionally, LTOT may be considered for those with PaO_2_ ≤ 60 mm Hg if they have signs of cor pulmonale, polycythemia (hematocrit ≥55%), or pulmonary hypertension. Although LTOT has shown significant effects in improving blood oxygen levels, its direct effects on exercise tolerance and daily function are limited. Therefore, oxygen therapy alone may be insufficient to address the multifaceted rehabilitation needs of COPD patients. At the same time, exercise-based rehabilitation is increasingly vital in COPD management, combining aerobic exercise, strength training, and breathing exercises to enhance muscle strength, endurance, cardiopulmonary function, and overall quality of life (6). However, the heterogeneous nature of COPD patient conditions means that a single rehabilitation model often fails to address the unique needs of all individuals. Consequently, personalized or multifaceted rehabilitation approaches may be necessary to effectively meet the diverse requirements of COPD patients.

The combined use of LTOT and exercise rehabilitation has attracted increasing attention as a synergistic strategy to enhance physical capacity, slow disease progression, and reduce the healthcare burden in COPD. Yet, variability in exercise protocols, differing in type, intensity, frequency, and duration, has led to inconsistent outcomes, posing challenges for clinical practice. This study therefore conducts a systematic meta-analysis to evaluate the effects of LTOT combined with exercise rehabilitation on exercise capacity, cardiopulmonary function, and quality of life in stable COPD, with the goal of informing more standardized and effective rehabilitation strategies.

## 2 Research content and methods

### 2.1 Sources and search methods for literature resources

The following electronic databases were used to do a thorough literature search: the Chinese Biomedical Literature Database (CBM), China National Knowledge Infrastructure (CNKI), ScienceDirect, EMBASE, Cochrane Library, VIP Full-Text Database, Wanfang Database, and PubMed. The search encompassed relevant Chinese and international journals, dissertations, conference papers, news articles, and manual retrieval sources, and was further supplemented by literature tracing methods. Studies eligible for inclusion were clinical control trials that evaluated the effects of oxygen therapy and exercise rehabilitation in patients with COPD.

The search strategy employed both free-text keywords and subject-specific terms, including but not limited to “oxygen therapy,” “exercise rehabilitation,” “COPD,” “exercise capacity,” “cardiopulmonary function,” and “quality of life.” The search period spanned from January 2010 to the present. The quality and relevance of the data gathered for the meta-analysis were guaranteed by eliminating duplicate research and choosing pertinent publications according to predetermined inclusion and exclusion criteria.

### 2.2 Inclusion criteria for literature

(1) Study type: randomized controlled trials (RCTs) and controlled cohort studies that investigated oxygen therapy and/or exercise rehabilitation as interventions for COPD patients.
(2) Study subjects: patients diagnosed with COPD; diagnostic criteria as described in relevant literature (7).
(3) Intervention measures: the study group (SG) received LTOT combined with exercise rehabilitation, while the control group (CG) received LTOT alone or exercise rehabilitation alone. Eligible rehabilitation programs included aerobic exercise, strength training, breathing training, or combinations thereof, with clearly defined duration.
(4) Reported one or more of the following outcome indicators: (1) 6-Min Walk Distance (6MWD); (2) blood gas indicators; (3) heart rate; (4) pulmonary function indicators; (5) quality of life.

### 2.3 Literature exclusion criteria

These criteria were used to determine which studies were not included in the meta-analysis:

(1) Non-controlled studies: studies without a parallel comparator group (e.g., single-arm designs).
(2) Incomplete data reporting: the reported data were incomplete or insufficient for analysis.
(3) Duplicate publications: studies that duplicated research content; in such cases, only the most recent study was included.
(4) Insignificant therapeutic effect evaluation: studies that did not include evaluable therapeutic outcomes relevant to this analysis.
(5) Review articles: publications that were review articles or related literature rather than original research.
(6) Case reports/case series: case reports and small case series without a parallel comparator.

### 2.4 Quality evaluation and data extraction

(1) Bias risk assessment of included studies: the evaluation was conducted using the “bias risk Assessment” technique suggested in the Cochrane Manual of Systematic Reviews 5.3. (2) Literature screening and data extraction: to maintain impartiality and reduce bias, two researchers independently carried out data extraction, literature screening, and quality rating. A third researcher was contacted to make the final judgment if the researchers were unable to achieve a consensus after discussing and resolving any disagreements. Microsoft Excel and Note Express literature management software made it easier to organize and extract research resources. For studies with incomplete data, the corresponding authors were contacted in order to acquire the necessary information. (3) The data extraction process encompassed the following elements: (1) basic information: author(s), year of publication, and number of participants; (2) intervention measures: details of the intervention plan and treatment course; (3) outcome indicators: 6MWD, blood gas indicators, heart rate, pulmonary function indicators, and quality of life, etc.

### 2.5 Statistical analysis

Using RevMan 5.3 software, the meta-analysis was completed in accordance with the Cochrane Collaboration's guidelines. For each outcome measure, including exercise endurance, blood gas indices, heart rate, lung function indices, and quality of life, the mean values and standard deviations for both the SG and the CG were extracted and input into RevMan 5.3. For outcomes measured using the same scale across all studies (e.g., 6MWD, PaO_2_, PaCO_2_, FEV1, FEV1/FVC), Mean difference (MD) with 95% confidence intervals (CIs) was calculated. For outcomes assessed using different measurement scales across studies (e.g., quality of life), Standardized Mean Difference (SMD) with 95% CIs was used. First, to ascertain if there was statistical heterogeneity, the Chi^2^ test was employed. Studies were considered homogenous if the *P*-value for the Chi^2^ test was more than 0.05 and the *I*^2^ statistic was less than 50%. In such cases, for the meta-analysis, a fixed-effect model was used. If the *P*-value from the Chi^2^ test was less than 0.05 and the *I*^2^ statistic was equal to or greater than 50%, demonstrating considerable heterogeneity, a random effects model (REM) was selected for the analysis. In instances where significant heterogeneity was detected (*P* < 0.05) and the sources of heterogeneity couldn't be identified; a meta-analysis was deemed inappropriate. Instead, a descriptive analysis was performed to summarize the findings qualitatively. Subgroup differences were examined using the Chi^2^ test for interaction. To account for clinical/intervention heterogeneity, subgroup analyses were prespecified. For exercise capacity (6MWD), studies were categorized by exercise regimen (mixed-modality vs. aerobic-only). For other outcomes (e.g., blood gases, pulmonary function, QoL), subgroup analyses were planned by exercise type, LTOT duration, or baseline hypoxemia severity (severe vs. non-severe/unclear). However, the analyses were not feasible due to the small number of studies and inconsistent reporting of intervention characteristics. To further examine the publication bias of the included literature, an inverted funnel plot was created. The asymmetry of the funnel plot was examined using Eggers' test. In cases where the *P*-value of this test was less than 0.1, the Trim and Fill method may be utilized to adjust the impact size of the potential publication bias and correct the funnel plot.

## 3 Results and analysis

### 3.1 The findings of literature search and the basic information of the included literature

The Preferred Reporting Items for Systematic Reviews and Meta-Analyses (PRISMA) standards were used for conducting the literature search. Initially, a total of 1,309 records were retrieved from computer databases. After removing duplicate studies, 934 unique records remained. Of these, 715 unnecessary studies were eliminated after these data were filtered based on their abstracts and titles. Subsequently, 423 studies were preliminarily included after excluding case reports, reviews, and uncontrolled studies.

**Figure 1:**
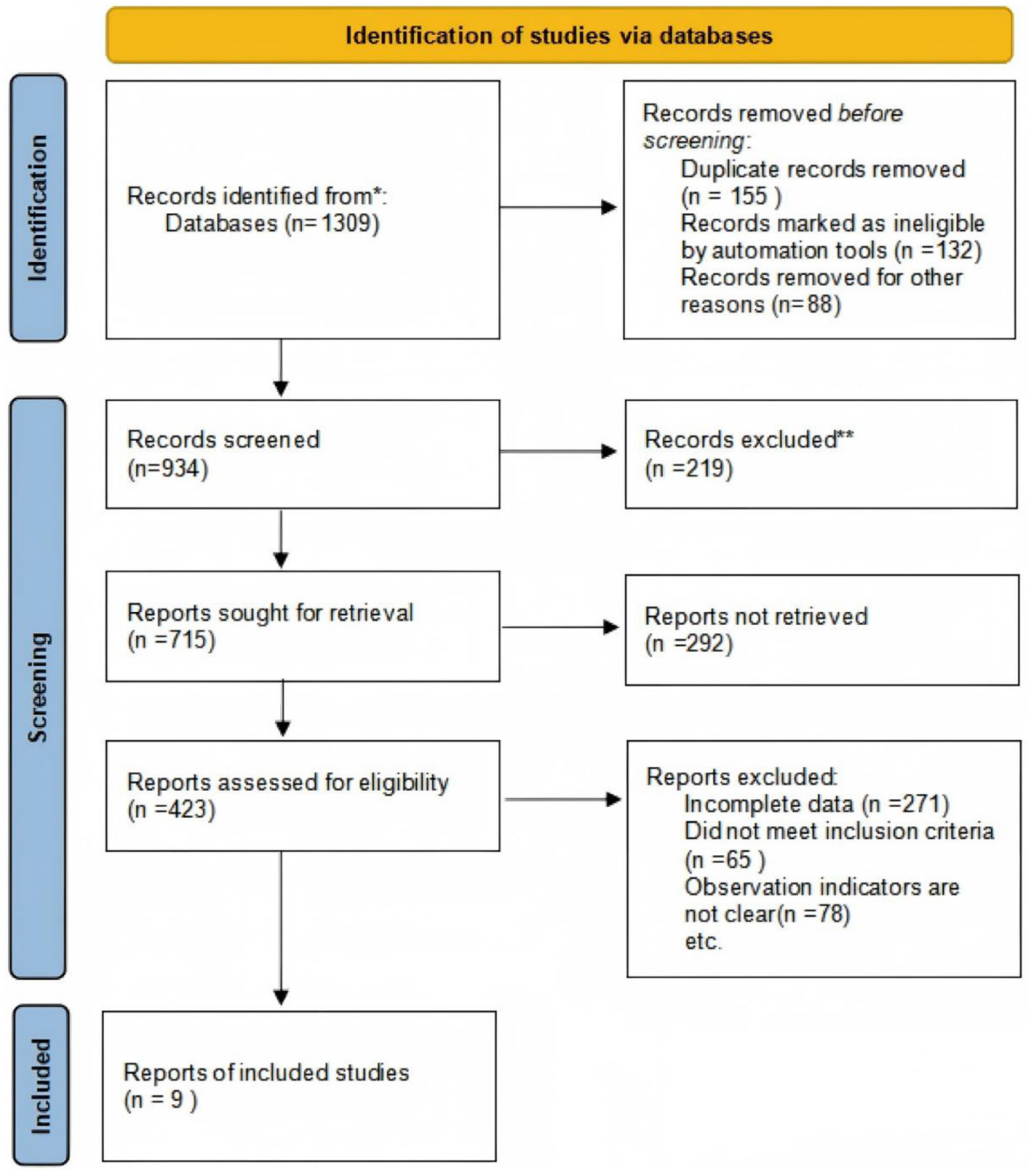
Literature screening flow chart.

The full texts of these 423 studies were meticulously reviewed to identify those with complete data and relevant primary outcome indicators. During this stage, 414 studies were excluded due to the absence of primary outcome measures. Nine clinical controlled trials, totaling 703 participants, were ultimately included in the meta-analysis after meeting all inclusion criteria. Figure 1 shows the literature screening flow chart. Table 1 displays the fundamental attributes of the included literature.

**Table 1:** Basic characteristics of literature.

**Included literature**	**Year of publication**	**Sample size**		**Intervention measure**		**Age**	**Outcome index**	**Literature quality evaluation score**
		**C**	**T**	**C**	**T**			
Jianmei et al. (8)	2021	49	49	Oxygen therapy	Oxygen therapy + pulmonary rehabilitation training (intervention for 3 months)	C: 67.16 ± 5.69 T: 68.58 ± 6.18	①④⑤	5
Yanli et al. (9)	2014	32	35	Oxygen therapy	Oxygen therapy + pulmonary rehabilitation training (intervention for 3 months)	C: 68.7 ± 7.4 T: 68.2 ± 7.1	①②③④⑤	6
Xuechen (33)	2024	48	48	Oxygen therapy	Oxygen therapy + pulmonary rehabilitation training (intervention for 8 weeks)	C: 60.23 ± 5.47 T: 60.31 ± 5.52	①②④	4
Renyi (34)	2019	65	65	Oxygen therapy	Oxygen therapy + lung rehabilitation training (Intervention for 6 months)	C: 62.31 ± 4.28 T: 62.03 ± 4.12	②④	4
Yiman (35)	2017	42	43	Oxygen therapy	Oxygen therapy + lung rehabilitation training (1 year intervention)	C: 64.2 ± 4.5 T: 62 ± 6.3	②④	4
Shangguang (36)	2020	25	25	Oxygen therapy	Oxygen therapy + lung rehabilitation training (1 year intervention)	C: 66.28 ± 5.17 T: 66.36 ± 5.18	②④	4
Weiping (37)	2022	50	50	Pulmonary rehabilitation training	Oxygen therapy + lung rehabilitation training (Intervention for 2 weeks)	C: 68.1 ± 2.4 T: 68.3 ± 2.1	②④	4
Marrara et al. (38)	2018	17	18	Oxygen therapy	Oxygen therapy + lung rehabilitation training (Intervention for 6 weeks)	C: 68.2 ± 8.5 T: 67.8 ± 8.9	①②③	5
Vitacca et al. (39)	2017	21	21	Oxygen therapy	Oxygen therapy + lung rehabilitation training (Intervention for 3 weeks)	C: 64.6 ± 11.7 T: 63.4 ± 11.8	①	4

① 6MWD; ② Blood gas index; ③ Heart rate; ④ Pulmonary function index; ⑤ Quality of life.

### 3.2 Evaluation of methodological quality of included literature

**Figure 2:**
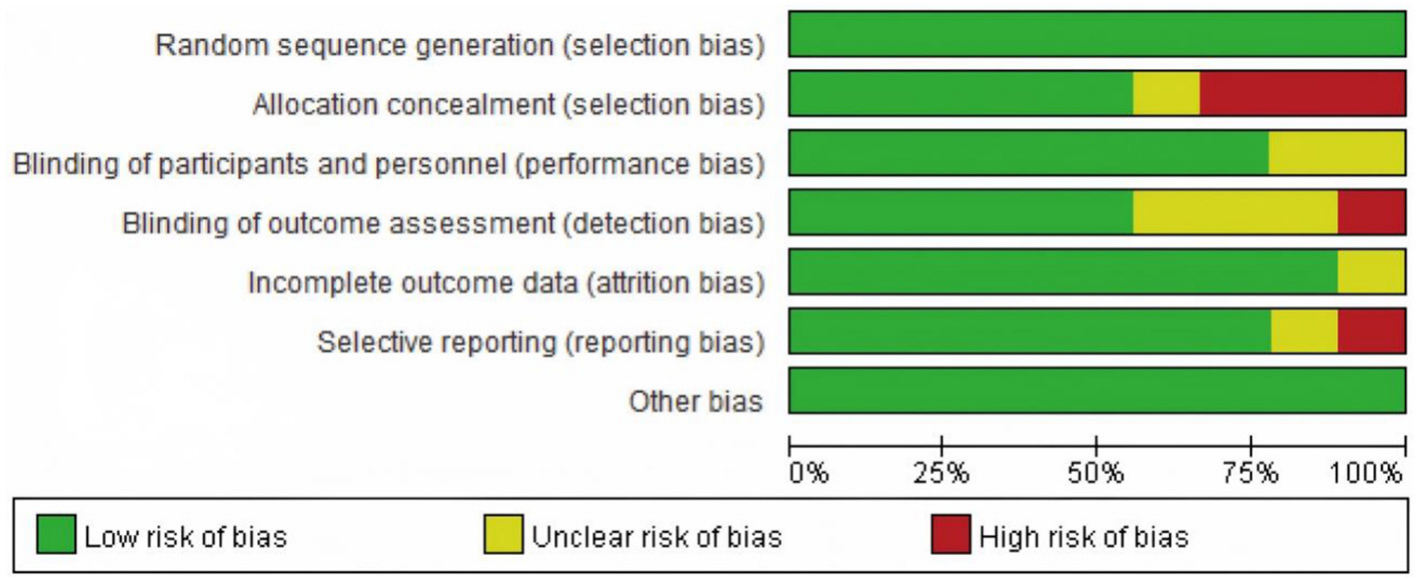
Risk bias diagram.

**Figure 3:**
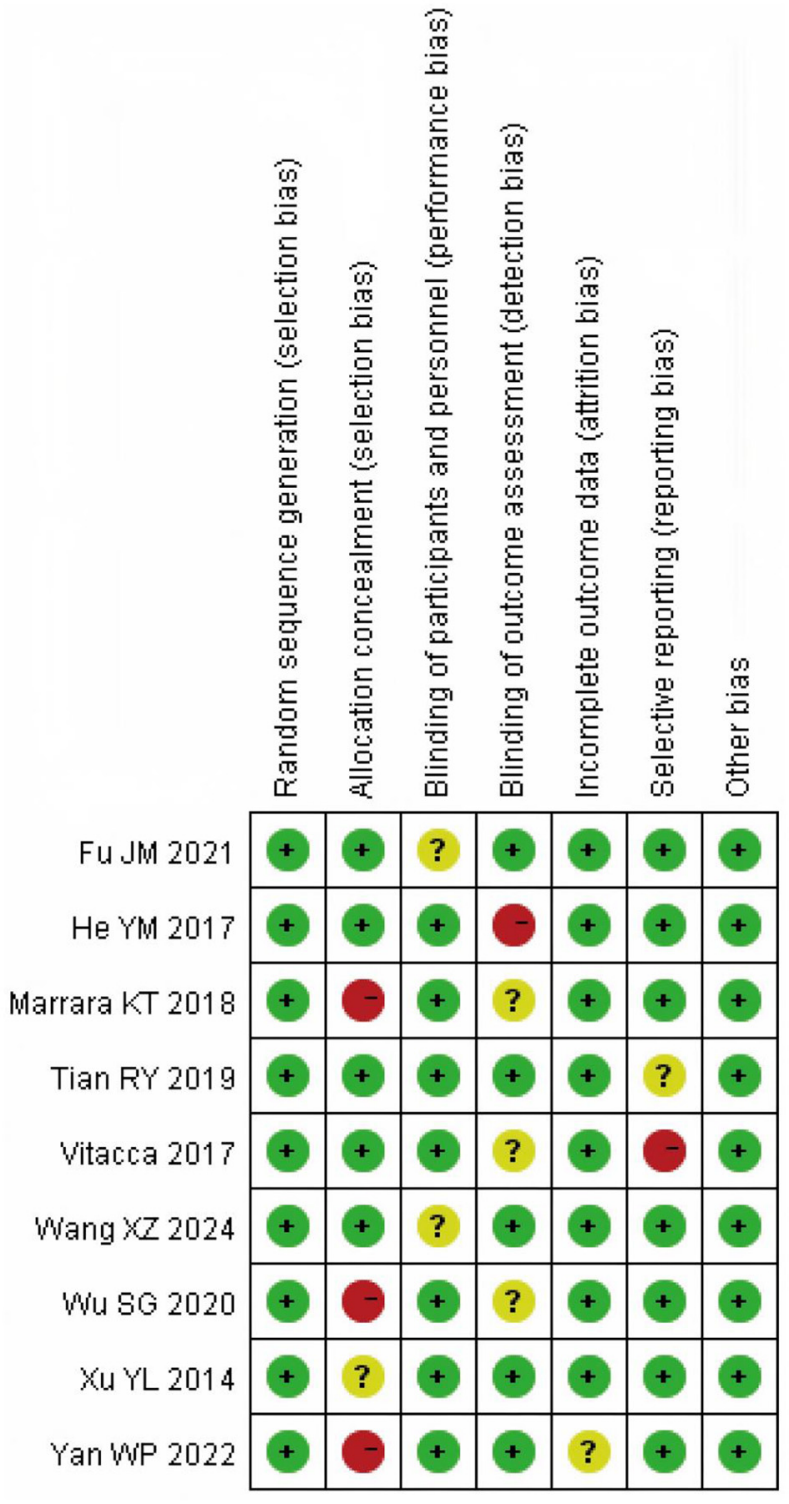
Summary of risk bias.

The methodological quality of the nine included studies was assessed using the Cochrane Risk of Bias Tool (version 5.3) across seven domains. Random sequence generation was low risk in 8/9 (88.9%) studies and unclear in 1/9 (11.1%). Allocation concealment was explicitly reported (low risk) in 2/9 (22.2%), unclear in 3/9 (33.3%), and high risk in 4/9 (44.4%) due to insufficient methodological detail. Blinding of participants/personnel (performance bias) was rated high in 3/9 (33.3%) and unclear in 6/9 (66.7%), reflecting the practical difficulty of blinding exercise/oxygen interventions and incomplete reporting. Blinding of outcome assessment (detection bias) was low risk in 4/9 (44.4%), unclear in 3/9 (33.3%), and high in 2/9 (22.2%). Attrition bias was low in 8/9 (88.9%) and unclear in 1/9 (11.1%). Selective reporting was low risk in 7/9 (77.8%), unclear in 1/9 (11.1%), and high in 1/9 (11.1%). Other bias was rated low risk in 9/9 (100%). These distributions correspond to the domain-level plots in Figures 2, 3.

### 3.3 Exercise endurance

**Figure 4:**
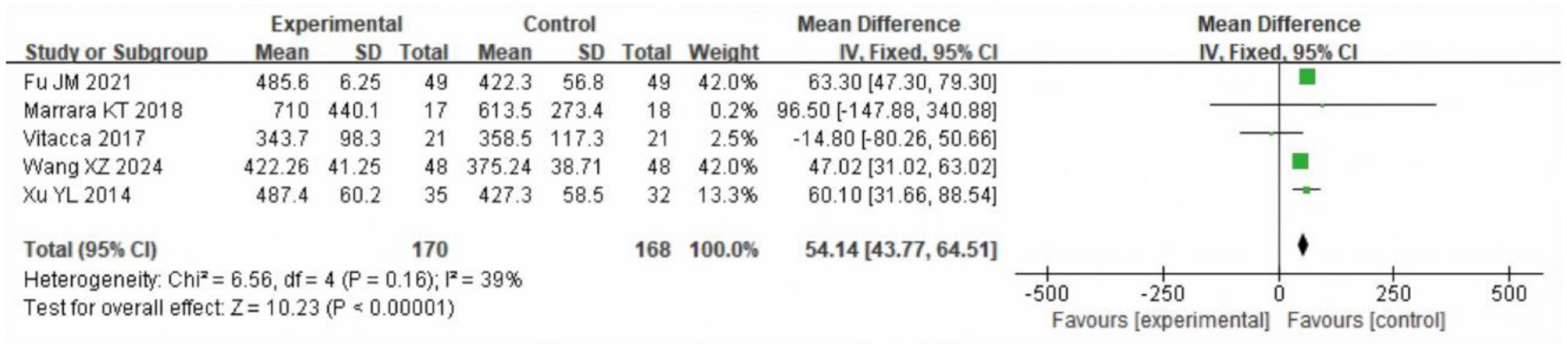
Forest analysis diagram of 6MWD comparison between the two groups after intervention. “Experimental” refers to the Study Group (SG) receiving LTOT + exercise rehabilitation; “Control” refers to the Control Group (CG) receiving either intervention alone.

**Figure 5:**
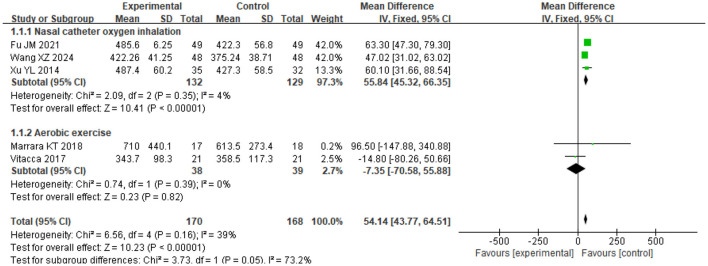
Forest plot analyzing the impact of different oxygen therapy modalities on the 6-Min Walk Distance (6MWD) in two groups of COPD patients. Experimental = SG; Control = CG.

The heterogeneity test showed χ^2^ = 6.56, df = 4, *P* = 0.16, *I*^2^ = 39%, indicating no significant heterogeneity. The fixed-effect model analysis (Figure 4) indicated that 6MWD in the SG was significantly longer than that in the CG after intervention (*P* < 0.05). When stratified by rehabilitation protocol, studies incorporating mixed exercise components (e.g., aerobic exercise, breathing training, or combined modalities) delivered alongside nasal catheter oxygen inhalation showed a mean improvement of 55.84 m (95% CI: 45.32–66.35) in 6MWD compared with the CG, while studies combining aerobic exercise with oxygen therapy showed no significant difference [−7.35 m (95% CI: −70.58 to 55.88)]. Across both approaches, the SG demonstrated longer 6MWD than the CG after intervention (*P* < 0.05; Figure 5).

### 3.4 Blood oxygen saturation

**Figure 6:**
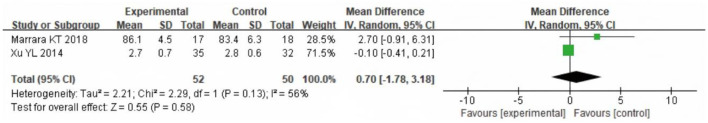
Comparison of blood oxygen saturation between the two groups after intervention. Experimental = SG; Control = CG.

A total of 102 participants from two studies reported SpO_2_. Heterogeneity was moderate (χ^2^ = 2.29, df = 1, *P* = 0.13; *I*^2^ = 56%). The REM analysis (Figure 6) showed no significant difference between groups (MD = 1.25%, 95% CI: −0.35 to 2.85; *P* = 0.13).

### 3.5 Arterial partial pressure of oxygen (PaO_2_)

**Figure 7:**
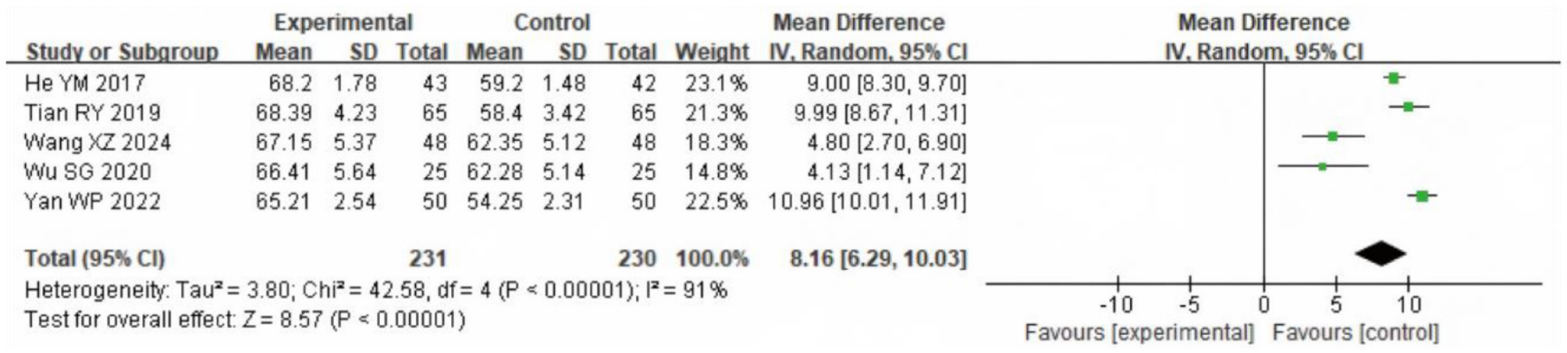
Forest analysis diagram of PaO_2_ comparison involving the two groupings after intervention. Experimental = SG; Control = CG.

Five studies including 461 participants evaluated PaO_2_. Heterogeneity was substantial (χ^2^ = 42.58, df = 4, *P* < 0.00001; *I*^2^ = 91%). The REM analysis (Figure 7) indicated that PaO_2_ was significantly higher in the SG (MD = 5.42 mmHg, 95% CI: 1.85–9.00; *P* = 0.003).

### 3.6 Arterial partial pressure of carbon dioxide (PaCO_2_)

**Figure 8:**
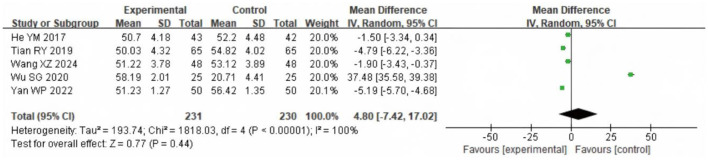
Forest analysis diagram of PaCO_2_ comparison involving the two groupings after intervention. Experimental = SG; Control = CG.

A meta-analysis was conducted on PaCO_2_ in the two groups after intervention, and a total of 461 samples were included into five literatures. The heterogeneity test results showed χ^2^ = 1,818.03, df =4, *P* < 0.00001, *I*^2^ = 100%, indicating that there was significant heterogeneity among the included study data. The REM analysis (Figure 8) showed no significant difference (MD = −0.86 mmHg, 95% CI: −3.20 to 1.48; *P* = 0.47).

### 3.7 Heart rate

**Figure 9:**
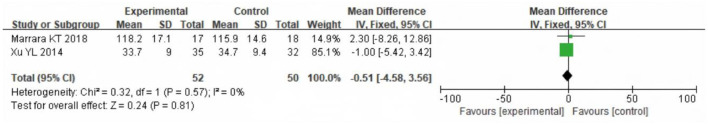
Forest analysis diagram of heart rate comparison between the two groups after intervention. Experimental = SG; Control = CG.

Two studies including 102 participants assessed heart rate. Heterogeneity was negligible (χ^2^ = 0.32, df = 1, *P* = 0.57; *I*^2^ = 0%), showing that the included study data showed no discernible heterogeneity. The fixed-effect model (Figure 9) showed no significant difference (MD = −1.15 beats/min, 95% CI: −4.32 to 2.02; *P* = 0.48).

### 3.8 FEV1 forced expiratory volume in 1 s (FEV1)

**Figure 10:**
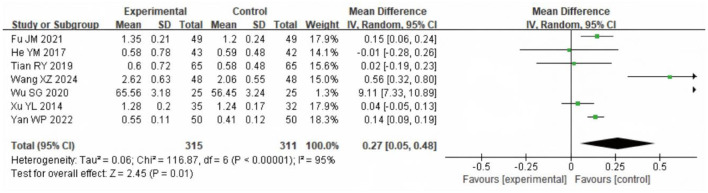
Forest analysis diagram for comparison of FEV1 values between the two groups after intervention. Experimental = SG; Control = CG.

A meta-analysis of FVE1 after intervention was conducted in the two groups, including seven articles with a total of 656 samples. The heterogeneity test results showed χ^2^ = 116.87, df = 6, *P* ≤ 0.00001, *I*^2^ = 95%, indicating considerable heterogeneity among the included research data. The REM analysis (Figure 10) showed that FEV1 was significantly higher in the SG (MD = 0.23 L, 95% CI: 0.08 to 0.38; *P* = 0.002).

### 3.9 FEV1/FVC [FEV1/forced vital capacity ratio (FVC)]

**Figure 11:**
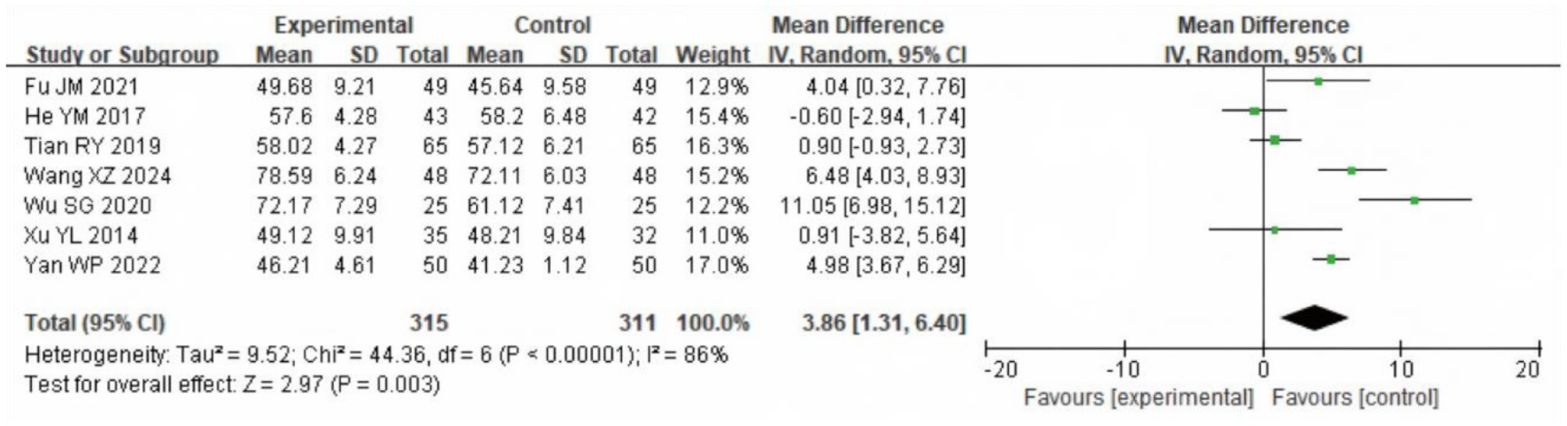
Forest analysis diagram comparing FEV1/FVC values between the two groups after intervention. Experimental = SG; Control = CG.

Seven studies with 656 participants assessed FEV1/FVC. Heterogeneity was substantial (χ^2^ = 44.36, df = 6, *P* < 0.00001; *I*^2^ = 86%). The REM analysis (Figure 11) showed that the SG had significantly higher FEV1/FVC values (MD = 3.86%, 95% CI: 1.31–6.40; *P* = 0.003).

### 3.10 Quality of life

**Figure 12:**
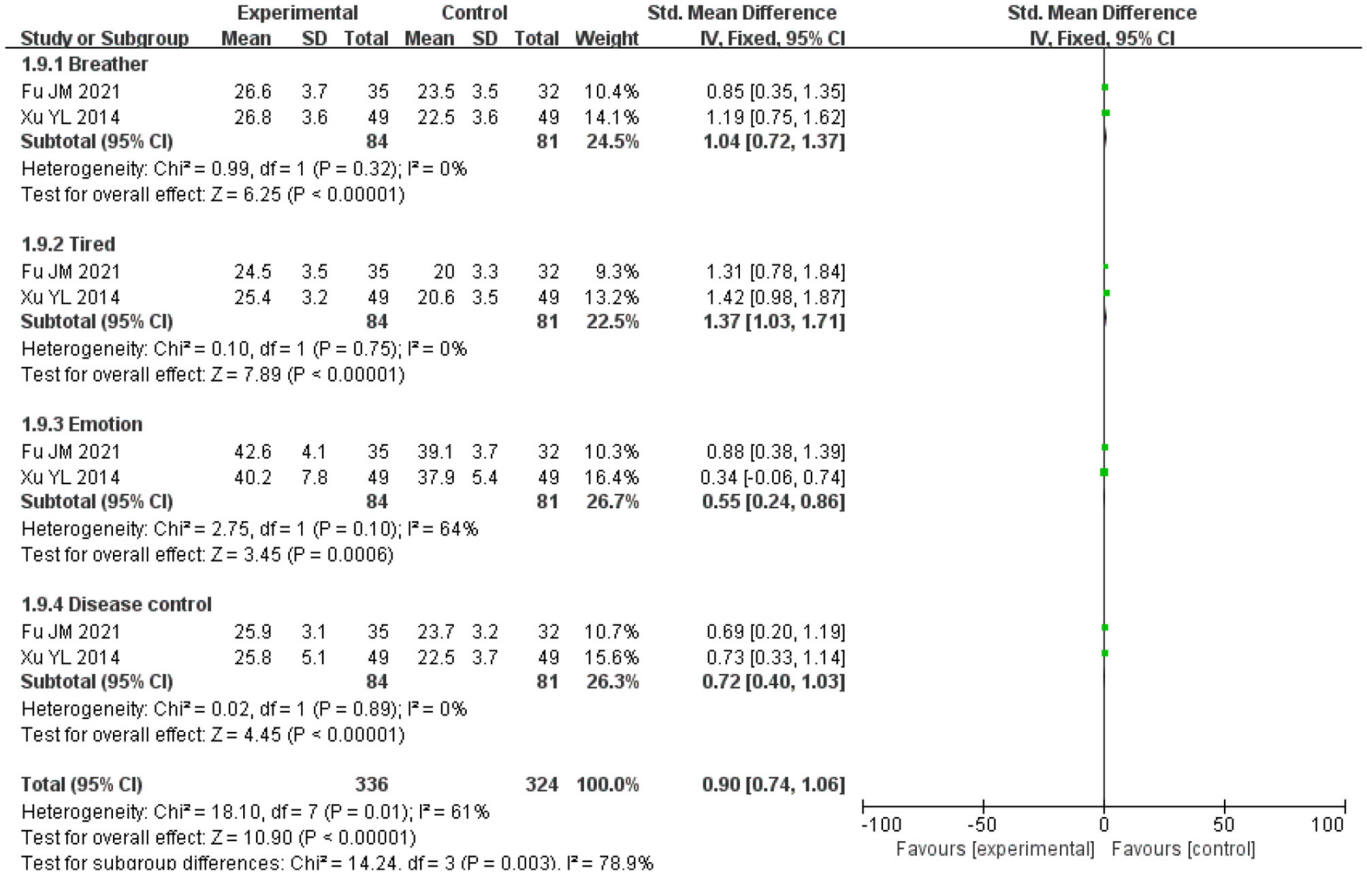
Forest analysis of the comparison of quality-of-life scores involving the two groupings after intervention. Experimental = SG; Control = CG.

A total of 165 patients from two trials [Jianmei et al. (8); Yanli et al. (9)] reported quality-of-life outcomes across four subdomains (breathing, tiredness, emotion, and disease control). The heterogeneity test showed χ^2^ = 9.80, df = 7, *P* = 0.20, *I*^2^ = 29%, indicating no significant heterogeneity. Pooled analysis using a fixed-effect model (Figure 12) demonstrated a SMD of 0.90 (95% CI: 0.74–1.06; *P* < 0.001), favoring the SG over the CG. This reflects a large effect size and suggests a clinically meaningful improvement in quality of life.

### 3.11 Publication bias analysis

**Figure 13:**
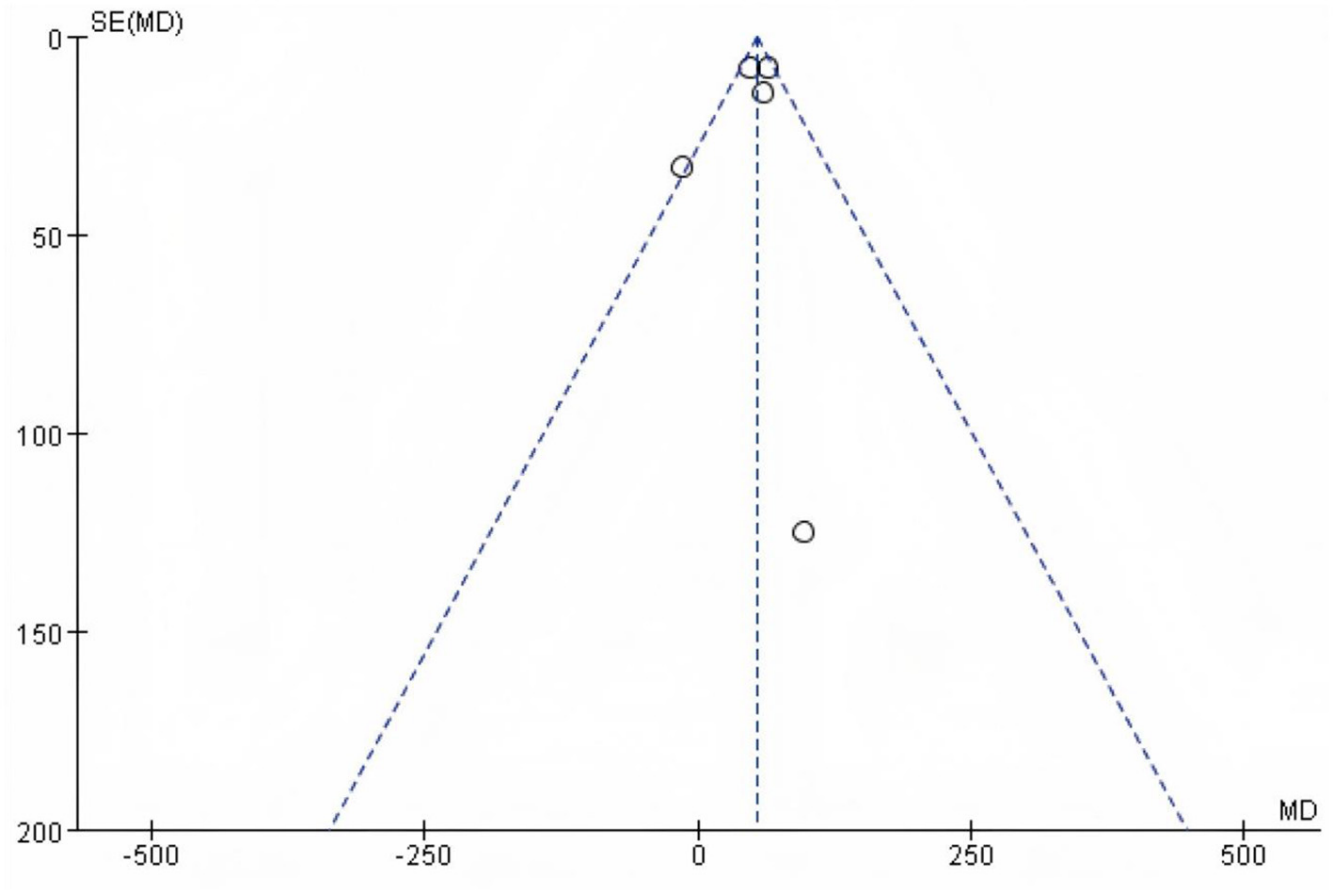
Funnel plot based on 6WMD.

**Figure 14:**
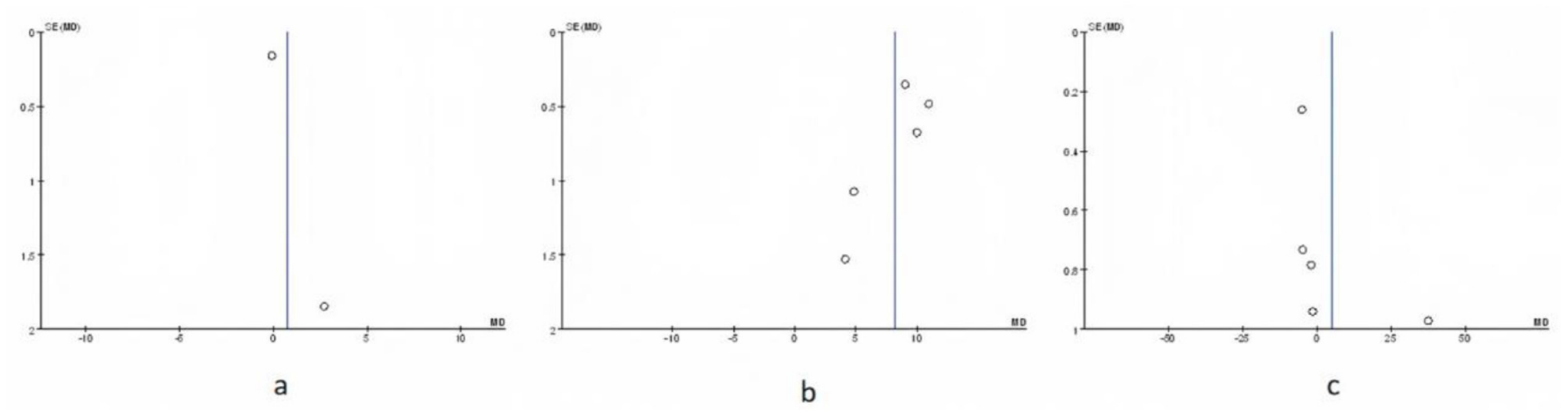
Funnel plot based on blood gas index. **(a)** Blood oxygen saturation; **(b)** PaO_2_; **(c)** PaCO_2._

**Figure 15:**
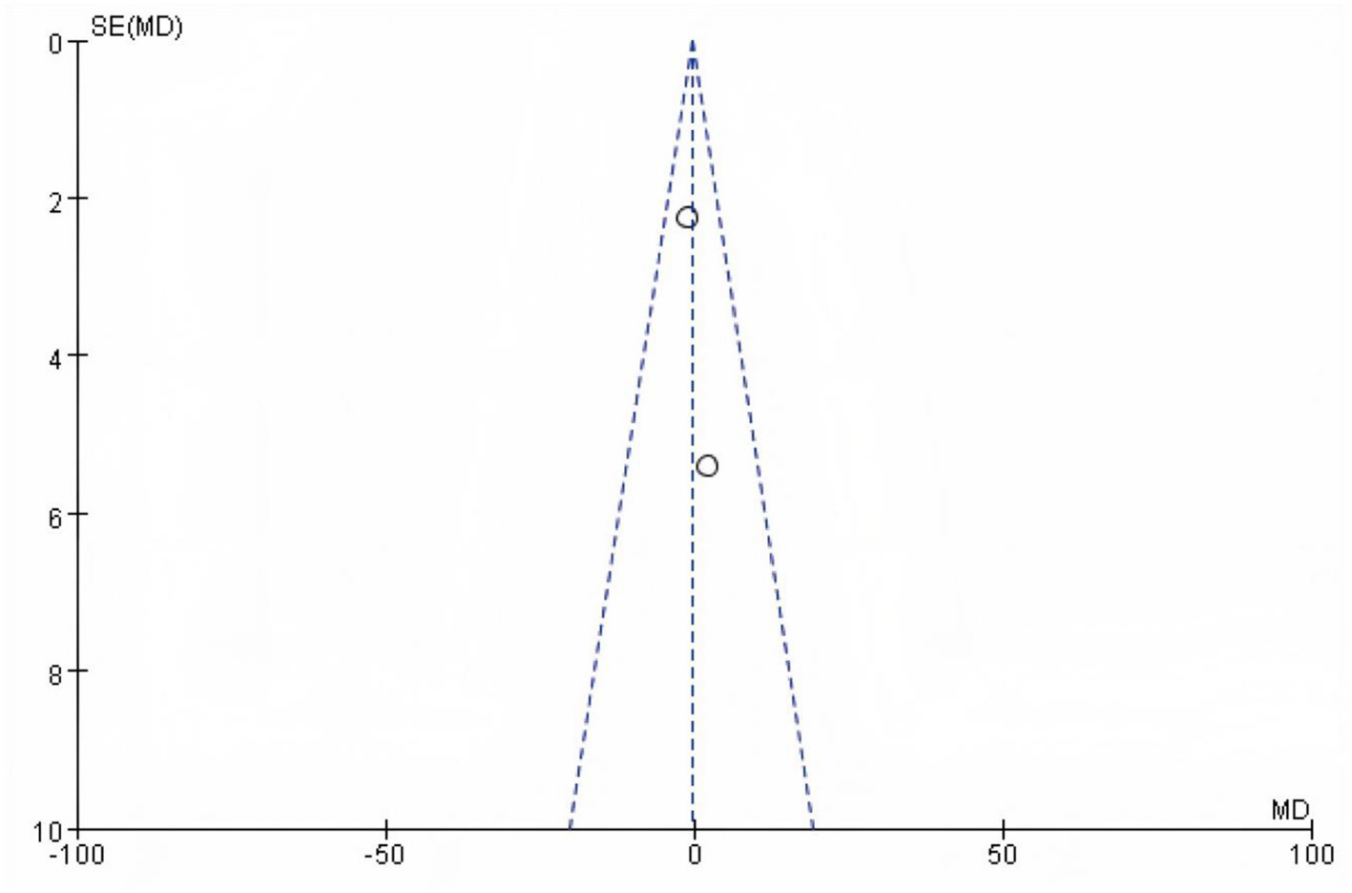
Funnel plot based on heart rate.

Funnel plots were prepared for each outcome (Figures 13–15; Supplementary Figures S1, S2). Across outcomes, the number of independent studies (k_studies) ranged from 2 to 7, and no outcome reached k_studies ≥10. For QoL, two trials reported CRQ domain scores; the funnel shows 7 domain-level estimates from these two studies, so k_studies = 2 and estimates = 7. In accordance with methodological guidance, Egger's regression was not performed for any outcome. Funnel plots are therefore descriptive only, and any apparent asymmetry should be interpreted cautiously given small k_studies and between-study heterogeneity.

## 4 Analysis and discussion

COPD is a common respiratory disease in clinical practice, with the characteristics of prolonged and difficult to cure, easy to repeat. According to relevant epidemiological study (10), the prevalence rate of COPD in adults over 20 years old in China is 8.6%, and that in adults over 40 years old can reach 13.7%, which has become one of the main disease factors threatening the life and health of middle-aged and elderly people in China. The course of COPD has two stages: acute exacerbation and stable stage. Compared with the acute stage, the severity of the condition of patients in stable stage is significantly reduced, but its potential risks cannot be ignored (11). Patients with stable COPD are susceptible to several physiological impairments, including reduced airway compliance, increased airway resistance, compromised ventilatory function, and diminished exercise endurance. These problems may result in hypoxemia and, in more extreme situations, respiratory failure, thereby posing significant risks to patients' lives and overall health (12, 13). Consequently, it is essential to implement scientifically grounded treatments and appropriate interventions at all stages of COPD. Such strategies aim to control the progression of the disease, prevent exacerbations, and improve the quality of life of patients (14, 15).

The improvement in exercise endurance is a key marker of rehabilitation efficacy in COPD (16). The patients often have problems such as limited respiratory function, decreased muscle strength, and chronic fatigue, which result in their exercise endurance being significantly lower than that of healthy people (17, 18). In our analysis, the 6MWD in the SG was consistently longer than in the CG by a pooled mean difference of 54.14 m (95% CI 43.77–64.51; *P* < 0.001; *I*^2^ = 39.1%; *k* = 5), a magnitude consistent with clinically meaningful improvement (*P* < 0.05). The gain was driven primarily by programs that delivered nasal-cannula oxygen during mixed-modality training (aerobic with breathing and/or strength work), which improved 6MWD by 55.84 m (95% CI 45.32–66.35); in contrast, aerobic-only exercise combined with oxygen did not show a clear advantage (MD −7.35 m, 95% CI −70.58 to 55.88). Both approaches were associated with improvement in walking distance, suggesting the beneficial impact of LTOT-supported rehabilitation across different structured exercise regimens. This finding may be attributed to the fact that exercise rehabilitation exercises enhance the muscle strength and peripheral blood flow in COPD patients, and improve the oxygen utilization efficiency of the whole body. In addition, the continuous oxygen supply of oxygen therapy supports the oxygen demand during exercise, thereby reducing the sense of dyspnea generated during exercise (19, 20).

COPD is primarily characterized by restricted airflow and impaired pulmonary ventilation. Consequently, improvements in pulmonary function indicators directly reflect the recovery of a patient's respiratory system (21). Commonly utilized pulmonary function metrics include Forced Vital Capacity (FVC), Forced Expiratory Volume in One Second (FEV1), and the FEV1/FVC ratio. These indicators serve not only as diagnostic and grading criteria for COPD but also play a vital part in evaluating the efficacy of rehabilitation interventions. Although the meta-analysis demonstrated a statistically significant improvement in the FEV1/FVC ratio following LTOT combined with exercise rehabilitation (WMD = 3.86%, 95% CI: 1.31–6.40; *P* = 0.003), this change lies near the lower threshold of clinical relevance. The FEV1/FVC ratio is widely used for diagnosis but is less sensitive for detecting short-term therapeutic effects in COPD. Current guidelines and expert consensus do not define a minimal clinically important difference (MCID) for this parameter. Prior studies suggest that changes smaller than 3% are unlikely to be clinically meaningful, whereas increases of 4%−5% or more may reflect relevant physiological improvement, particularly when accompanied by gains in FEV1 or quality of life (22–24). Our observed effect approaches this threshold, suggesting possible clinical importance, but should be interpreted alongside other outcome measures. Moreover, significant improvements in pulmonary function were observed following the combined intervention of LTOT and exercise rehabilitation. This enhancement is likely attributable to the synergistic effects of these treatments in promoting lung function recovery. Specifically, LTOT improves tissue oxygenation by providing sufficient oxygen, thereby alleviating breathing difficulties and reducing the cardiorespiratory burden caused by hypoxia (25, 26). Concurrently, exercise rehabilitation enhances the strength and endurance of respiratory muscles, which improves lung ventilation efficiency and further facilitates the recovery of pulmonary function (27).

For blood gases, PaO_2_ was higher in the study group, but with substantial between-study heterogeneity (χ^2^ = 42.58, df = 4; *I*^2^ = 91%; *P* < 0.00001; *k* = 5), whereas PaCO_2_ showed no statistically significant between-group difference with very high heterogeneity (*I*^2^ = 100%; *k* = 5). SpO_2_ likewise did not differ significantly (*k* = 2). These very high *I*^2^ values indicate that true effects vary across studies, so a single average effect should be interpreted cautiously. Plausible contributors to variability include differences in oxygen modality, oxygen titration and flow targets, program duration, and baseline hypoxemia severity across trials. In addition, structural lung damage in COPD (e.g., airway stenosis and alveolar destruction) limits gas exchange capacity, making large and uniform improvements in PaO_2_ or PaCO_2_ less likely in the short term (28–30). PaCO_2_ stability is also influenced by systemic factors such as metabolic status and respiratory drive (31, 32). These factors likely underlie the null effects and high heterogeneity, emphasizing the need for standardized protocols and larger trials to define the impact of LTOT plus rehabilitation on blood gases. Similarly, quality of life showed a pooled effect size of SMD = 0.90, suggesting a potentially large and clinically relevant improvement. However, this finding is derived from only two studies (*n* = 165), and should therefore be interpreted with caution. Compared with lung rehabilitation training alone, LTOT combined with exercise rehabilitation was associated with effective outcomes. This combination helps reduce fear and anxiety related to hypoxia, alleviates breathing difficulties, enhances exercise compliance, and ultimately improves the quality of life in COPD patients. Although there are some differences in exercise rehabilitation exercise standards and exercise time among the literatures included in this study, the overall results suggest that LTOT combined with exercise rehabilitation may be associated with improvements in exercise capacity, cardiopulmonary function, and quality of life of COPD findings. These benefits appeared across different program types, including mixed-modality and aerobic-focused protocols, indicating a consistent trend despite variability. However, given the small number of trials, heterogeneity in protocols, and overall moderate methodological quality, these findings should be regarded as tentative, and further high-quality, standardized studies are needed to confirm the robustness and generalizability of the effects.

This review is limited by the moderate methodological quality (grade B) of the included trials and by incomplete reporting in key domains. In particular, allocation concealment was adequately reported in 2/9 studies (22%), and blinded outcome assessment in 4/9 (44%), while performance bias was high or unclear in all studies given the practical challenges of blinding exercise/oxygen interventions; these features introduce potential selection and performance/detection bias that may influence effect estimates. Between-study heterogeneity was substantial for several outcomes, most notably PaCO_2_ (*I*^2^ ≈ 100%), and likely reflects variation in oxygen-delivery approach and titration, baseline hypoxemia, and rehabilitation dose/content (type, intensity, and duration). Exploratory subgroup analysis was possible for 6MWD, which suggested benefits in mixed-modality programs with LTOT, but subgroup analyses for other outcomes (e.g., PaO_2_, FEV1, QoL) were not feasible due to the small number of studies and inconsistent reporting. Future trials should incorporate pre-specified subgroup analyses to clarify these differences. Quality of life outcomes were available from only two small regional trials [Jianmei et al. (8); Yanli et al. (9)]. These were non-indexed studies with limited methodological reporting, which substantially reduces certainty and generalizability of the pooled QoL estimate. Assessment of small-study effects was also constrained: no outcome met *k* ≥ 10, so formal asymmetry tests (e.g., Egger) were not performed and funnel plots are descriptive only. Moreover, two trials [Jianmei et al. (8); Yanli et al. (9)] were published in non-indexed regional journals, which may limit external generalizability. Finally, this review was not prospectively registered in PROSPERO or another registry. While this limits transparency, we adhered closely to PRISMA guidelines and predefined our eligibility criteria, search strategy, and analytic approach to minimize bias. Taken together with generally small sample sizes, moderate methodological quality, and reliance on two non-indexed regional trials, these factors reduce certainty around pooled effects. Future research should prioritize multicenter randomized controlled trials with rigorous allocation concealment and blinded outcome assessment, prospective protocol registration (e.g., PROSPERO), and standardized oxygen delivery targets and rehabilitation protocols. In addition, adequately powered studies should specifically evaluate outcomes with high heterogeneity (e.g., PaCO_2_, FEV1) and confirm the tentative quality-of-life findings, which currently rest on only two small studies. Such work is needed to refine both the magnitude and the generalizability of benefit.

In summary, LTOT combined with exercise rehabilitation was associated with improvements in pulmonary function, exercise capacity, and quality of life in patients with COPD. While these findings are encouraging, they should be interpreted with caution due to the limited number of studies, variability in intervention protocols, and the moderate methodological quality of the included trials. Future large-scale, rigorously designed RCTs are needed to confirm these benefits and define optimal intervention.

## Data Availability

The raw data supporting the conclusions of this article will be made available by the authors, without undue reservation.
