# Supplementary material for: A meta-analysis of the effects of long-term oxygen therapy combined with exercise rehabilitation on exercise capacity, cardiopulmonary function, and quality of life in patients with COPD
Source: Front Med (Lausanne). 2025 Sep 22;12:1640084. doi: 10.3389/fmed.2025.1640084 (PMC12497624; doi:10.3389/fmed.2025.1640084)
Supplement: Supplementary file 1 [file Data_Sheet_1.docx]

**SupplementaryFigures
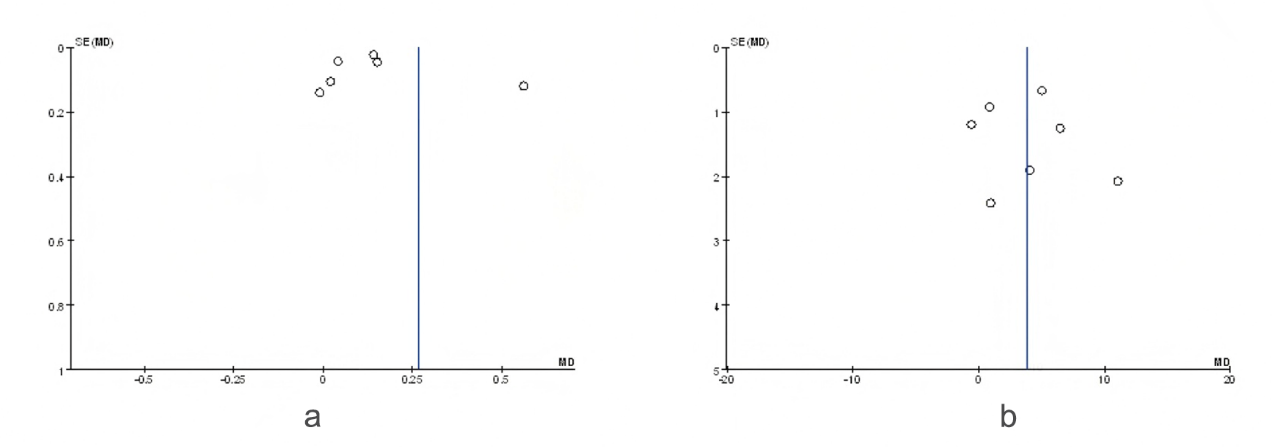
**

**Fig. S1.** Funnel plot based on pulmonary function index. Note: a: FEV1; b: FEV1/FVC.

**
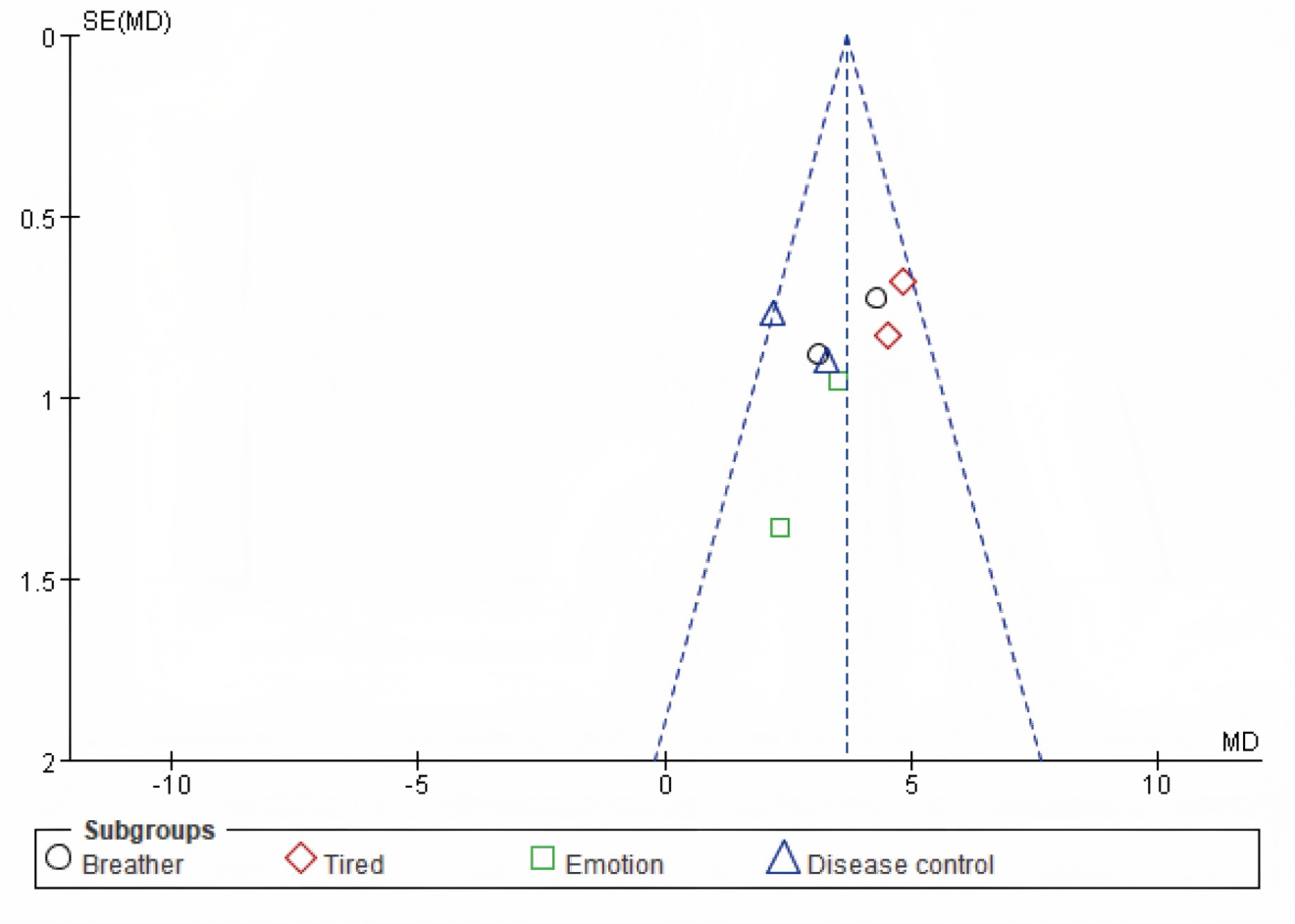
**

**Fig. S2.** Funnel plot based on quality-of-life score. Note: a: FEV1; b: FEV1/FVC.
